# Analysis of the clinical effect of dexmedetomidine hydrochloride for sedation before cosmetic suturing of emergency facial trauma in children

**DOI:** 10.3389/fsurg.2026.1814772

**Published:** 2026-04-24

**Authors:** Wenbin Gao, Xianpu Song, Yanhua Feng, Chuo Liu

**Affiliations:** Hebei Children's Hospital, Emergency Surgery Department of Hebei Children's Hospital, Hebei Provincial Clinical Research Center for Child Health and Disease, Shijiazhuang, China

**Keywords:** cosmetic suture, dexmedetomidine hydrochloride, facial trauma, pediatric emergency, preoperative sedation

## Abstract

**Objective:**

To explore the clinical efficacy and safety of dexmedetomidine hydrochloride for preoperative sedation in children undergoing emergency facial trauma cosmetic suturing.

**Methods:**

A total of 200 children with facial trauma admitted to the Emergency Surgery Department of our hospital from January to June 2025 were retrospectively enrolled and assigned to two groups for different sedation interventions. The study group received preoperative intranasal dexmedetomidine hydrochloride spray, while the control group received routine comfort care. The UMSS Sedation Scale was used to assess sedation level objectively, and rescue dosing protocols were implemented for sedation failure. Statistical analyses were performed using SPSS 30.0 software, with a two-tailed *P*-value ≤ 0.05 and 95% confidence intervals (CIs) considered statistically significant.

**Results:**

A total of 100 cases were included in both the study group and the control group, with no baseline differences between the two groups (*P* > 0.05). The sedation failure rate in the study group was 8.0% (8/100), and all 8 cases achieved effective sedation after rescue dosing with one additional spray (15*μ*g for 10.7–19.4 kg, 25 μg for 19.4–28.0 kg) intranasal dexmedetomidine hydrochloride; no sedation failure was observed in the control group. No serious adverse reactions were observed in either group. Preoperative and intraoperative cooperation in the study group was significantly better than that in the control group, and the incidence of agitation was significantly lower (*P* < 0.01), 95%CI: 0.0–0.2. No serious adverse reactions were observed in either group. Preoperative heart rate, respiratory rate, oxygen saturation, and mean arterial pressure were comparable between the two groups (*P* > 0.05). Intraoperatively, the study group maintained more stable vital signs, with significantly lower heart rate and mean arterial pressure than the control group (*P* < 0.05), 95%CI for heart rate: 8.2–15.7; 95%CI for mean arterial pressure: 5.1–10.3, all within normal physiological ranges.

**Conclusion:**

Preoperative intranasal dexmedetomidine hydrochloride is an effective and safe regimen for improving intraoperative cooperation, stabilizing vital signs, reducing agitation, and enhancing cosmetic suture quality and family satisfaction in children with emergency facial trauma.

## 1 Introduction

Children aged 2–6 years old are prone to facial trauma due to accidental collisions and falls due to their immature cognitive abilities and self-protective abilities. Improper treatment of facial trauma will leave obvious scars, which not only affects the appearance, but also has long-term negative psychological effects on the child (1, 2). As parents have increased aesthetic demands for trauma repair, emergency cosmetic suturing has become the preferred treatment method for children with facial trauma (3).

Compared with traditional sutures, the key to emergency cosmetic sutures is the precise alignment of the wound, using 6-0 or 7-0 sutures, through fine layered tension reduction, alignment sutures, and fine micro-mark alignment on the epidermis (4). Precise anatomical reduction, tension dispersion and minimally invasive operations can restore the original microstructure of the wound to the greatest extent, and can restore the microstructure of the original wound tissue to the greatest extent, thereby reducing the obvious degree of scars (5, 6).

However, children with facial trauma often show crying, irritability, and poor cooperation due to fear, pain, and tension during the treatment process, which prolongs the diagnosis and treatment time, reduces the accuracy of suturing operations, and ultimately damages the aesthetic healing effect of the wound (7). In addition, restlessness can also lead to increased blood pressure, increased wound bleeding, and unclear surgical field of view, making the operation more difficult. Therefore, safe and effective sedation to improve children's intraoperative cooperation was a key clinical issue that needs to be solved for cosmetic suturing of emergency facial trauma.

Dexmedetomidine hydrochloride was a highly selective *α*2-adrenergic receptor agonist. It inhibits the release of neurotransmitters in the presynaptic membrane by binding to *α*2A and *α*2B subtype receptors, and exerts sedative, analgesic and anxiolytic effects (8). Intranasal administration of dexmedetomidine had the advantages of non-invasiveness, rapid onset of action, and high acceptability, and showed good application potential in pediatric preoperative sedation (9). However, its specific clinical effect and safety in the special scenario of cosmetic suturing of emergency facial trauma in children needed to be further verified. This randomized controlled trial aimed to evaluate the effectiveness and safety of intranasal dexmedetomidine hydrochloride for sedation before cosmetic suturing of emergency facial trauma in children, and provided an evidence-based reference for clinical practice.

## 2 Materials and methods

### 2.1 Study design and ethical approval

This retrospective trial was approved by the Ethics Committee of Hebei Children's Hospital, and the research was conducted in strict compliance with the relevant requirements of the Declaration of Helsinki and Good Clinical Practice Guidelines. The legal guardians of all participating children signed written informed consent forms.

### 2.2 Determination of sample size

This was a retrospective cohort study. The electronic medical record system of our hospital was searched to identify children with facial trauma admitted to the Emergency Surgery Department from January to June 2025. After screening according to the inclusion and exclusion criteria, a total of 200 eligible children were finally enrolled. Retrospective randomization was used for group assignment, and the UMSS Sedation Scale (score 1–5) was applied to all children for objective sedation assessment at 5, 10, and 15 min after intervention.

### 2.3 Clinical data and research subjects

Inclusion criteria: 1. 2 to 6 years old; 2. Diagnosed with acute facial soft tissue trauma requiring emergency cosmetic suturing; 3. No heart, liver, kidney or other important organ diseases, and no congenital genetic diseases; 4. No history of allergies to dexmedetomidine hydrochloride, lidocaine or related suture materials; 5. With complete fasting and water deprivation 30 min before surgery; 6. Legal guardian fully understood the research plan and signed an informed consent form.

Exclusion criteria: 1. Severe trauma combined with damage to the brain, orbit and other organs; 2. Abnormal vital signs on admission (shock, tachycardia, hypoxemia, etc.); 3. History of neurological or mental illness; 4. Receiving other sedative or analgesic drugs within 24 h before admission; 5. Unable to complete studies due to guardian refusal or other reasons.

### 2.4 Randomization, grouping and blinding

From the clinical cases that have been collected in our hospital that meet the inclusion and exclusion criteria, a computer-generated random number table method was used for group assignment. Random sequences were generated and maintained by an independent statistician who was not involved in clinical operations and data collection. Because the intervention in this study was intranasal administration, the medical staff and nursing staff who performed the surgery could not be blinded; the statistician responsible for data analysis and the intraoperative cooperation assessor were blinded, and were not informed of the grouping status until the completion of the data analysis. Rescue dosing protocol for sedation failure: If the UMSS Sedation Scale score was <3 at 15 min after intranasal dexmedetomidine administration, one additional spray (15μg for 10.7–19.4 kg, 25 μg for 19.4–28.0 kg) was administered intranasally, and the sedation effect was re-evaluated 10 min later. A total of 8 cases in the study group had initial sedation insufficiency (UMSS score < 3) at 15 min after administration, and both achieved effective sedation (UMSS score 3–5) after rescue dosing according to the above protocol; no sedation failure or rescue dosing was required in the control group.

### 2.5 Methods

#### 2.5.1 Study group

Ten minutes before the operation, dexmedetomidine hydrochloride (Shanghai Hengrui Pharmaceutical Co., Ltd., Shanghai, China; specifications: 1 mL: 300 μg, national drug approval number: H20230025) was administered by intranasal spray, and the family members and medical staff simultaneously provided comfortable care. The dose was administered intranasally according to the child's body weight: For children weighing 10.7 kg ≤ body weight < 19.4 kg, 1 mL of the 300 μg formulation was administered; For children weighing 19.4 kg ≤ body weight < 28 kg, 1 mL of the 500 μg formulation was administered. Sedation level was monitored using the UMSS Sedation Scale at 5, 10, and 15 min after spray administration, and rescue dosing was implemented immediately for children with a UMSS score < 3 at 15 min according to the pre-set protocol one additional spray (15μg for 10.7–19.4 kg, 25 μg for 19.4–28.0 kg).

#### 2.5.2 Control group

The children in the control group only received routine comfort from family members and medical staff before surgery, and did not use any sedative drugs. If the child still did not cooperate well, family members would help to fix the head and limbs to ensure that the range of facial movement was minimized. The UMSS Sedation Scale was also used for cooperation assessment in the control group, with the same evaluation time points as the study group.

The surgical procedure consisted of the following steps: A cosmetic suturing pathway was designed tailored to the wound condition. For the deep tissue, layered interrupted tension-reducing suturing was performed with 6-0 absorbable sutures (REF:C0068006; B.Braun Surgical, S.A., Rubí, Spain) to ensure good alignment of the skin edges; the epidermal layer was then sutured with 7-0 non-absorbable sutures (REF:EH7222H; Ethicon Inc., Somerville, NJ, USA) for secondary tension reduction and precise edge alignment. All children in both groups were observed for 30 min postoperatively.

### 2.6 Study indicators

The heart rate, respiratory rate, blood oxygen saturation and mean arterial pressure of the two groups of children before and after surgery were monitored and recorded. At the same time, intraoperative cooperation was evaluated combined with the UMSS Sedation Scale, and the classification criteria were as follows:

1. No restlessness (UMSS score 5): quiet, no crying, no physical resistance, can cooperate with cosmetic suture surgery; 2. Slightly restless (UMSS score 4): crying but no physical resistance, can reluctantly cooperate with cosmetic suture surgery; 3. Moderate agitation (UMSS score 3): crying accompanied by physical resistance, but routine suture surgery can be performed under the control of family members and medical staff; 4. Severe agitation (UMSS score 1–2): severe crying, obvious physical resistance, beyond the control of family members and medical staff, making routine suturing surgery impossible.

To ensure the consistency and objectivity of sedation assessment, the validated 5-point University of Michigan Sedation Scale (UMSS) for pediatric sedation was adopted, with the following criteria (10)

1. Score 1: Wide awake, alert, active, and uncooperative with the procedure;
2. Score 2: Awake, restless or anxious, requiring frequent verbal reassurance to maintain cooperation;
3. Score 3: Awake, calm, responsive to verbal commands, with occasional mild resistance;
4. Score 4: Drowsy, easily aroused by verbal commands or light touch, able to cooperate with simple instructions;
5. Score 5: Asleep, unarousable with deep tactile stimulation or painful stimuli.

“No restlessness (UMSS score 5)” and “slight restlessness (UMSS score 4)” were classified as effective cooperation for cosmetic sutures, and “moderate agitation (UMSS score 3)” and “severe agitation (UMSS score 1–2)” were classified as invalid cooperation. In addition, the occurrence of adverse reactions such as nausea, vomiting, respiratory depression, and bradycardia in children was recorded. 95% CIs were calculated for all key outcome indicators including effective cooperation rate, agitation incidence rate and adverse reaction incidence rate.

Key outcome indicators: Effective cooperation rate, agitation incidence, sedation failure rate, and changes in vital signs; 95% CIs were calculated for all key rates and measurement data.

### 2.7 Statistical methods

SPSS 30.0 statistical software (SPSS Inc., Chicago, IL, USA) was used for data analysis. Measurement data were expressed as mean ± standard deviation, and the independent-samples t-test was used for comparison between groups, and 95% CIs were calculated for the mean difference between groups. Enumeration data were expressed as rates or percentages, and the *χ*^2^ test was used for intergroup comparison; 95% CIs were calculated for all rates using the binomial distribution method. Ranked data were analyzed using the rank-sum test. A value of *P* ≤ 0.05 was considered statistically significant.

## 3 Results

### 3.1 Comparison of vital signs indicators between the two groups of children

As shown in Table 1, there were no statistically significant differences in the baseline data, including gender, age, body weight, wound conditions, preoperative heart rate and mean arterial pressure, between the two groups (*P* > 0.05) 95%CI for preoperative HR: −6.7 to 9.1; 95%CI for preoperative MAP: −2.7 to 3.5, indicating good comparability.

**Table 1:** Comparison of baseline data between the two groups of children.

Characteristics	Study group (*n* = 100)	Control group (*n* = 100)	*P* value
Gender (male/female)	56/44	52/48	>0.05
Age (years)	4.0 ± 0.82	3.9 ± 0.91	>0.05
Weight (kg)	15.2 ± 1.73	14.9 ± 1.85	>0.05
Wound length (cm)	3.3 ± 0.81	3.4 ± 0.76	>0.05
Wound location (midface/lateral face)	65/35	61/39	>0.05
Preoperative HR (beats/min)	112.3 ± 10.5	111.1 ± 9.8	>0.05
Preoperative MAP (mmHg)	82.3 ± 6.5	81.9 ± 6.1	>0.05

As can be seen from Table 2, there were no statistically significant differences in the preoperative heart rate, respiratory rate, blood oxygen saturation and mean arterial pressure between the two groups of children (*P* > 0.05) 95%CI for HR: −6.7 to 9.1; 95%CI for RR: −0.5 to 0.9; 95%CI for MAP: −2.7 to 3.5; 95%CI for oxygen saturation: −0.6 to 1.0. During the operation, the heart rate, respiratory rate and mean arterial pressure of the study group were significantly lower than those of the control group (*P* < 0.05) 95%CI for HR: −14.7 to −8.1; 95%CI for RR: −3.9 to −2.5; 95%CI for MAP: −5.8 to −3.2, and all indicators were within the normal physiological range; the blood oxygen saturation of the two groups remained at normal levels throughout the entire process, and there were no significant differences between the groups (*P* > 0.05) 95%CI: −0.6 to 1.0.

**Table 2:** Comparison of vital signs indicators between the two groups of children.

Group	Time point	HR (beats/min)	RR (breaths/min)	Oxygen saturation (%)	Mean arterial pressure (mmHg)
Study group (*n* = 100)	Preoperative	112.3 ± 10.5	24.4 ± 3.3	98.4 ± 1.3	82.3 ± 6.5
	Intraoperative	97.1 ± 8.9*	20.5 ± 2.9*	97.9 ± 1.4	75.8 ± 5.6*
Control group (*n* = 100)	Preoperative	111.1 ± 9.8	24.2 ± 3.4	98.2 ± 1.5	81.9 ± 6.1
	Intraoperative	108.5 ± 9.6	23.7 ± 3.2	98.0 ± 1.2	80.3 ± 6.2

Compared with the control group in the same period, *P* < 0.05.

*Statistically significant difference compared with the control group at the same time point (*P* < 0.05).

### 3.2 Comparison of intraoperative cooperation and incidence of agitation between the two groups of children

As shown in Table 3, the intraoperative cooperation of the children in the study group was significantly better than that of the control group, and the incidence of agitation was significantly lower than that of the control group (*P* < 0.01), 95%CI for effective cooperation rate difference: 19.2% to 40.8%; 95%CI for agitation incidence difference: −40.8% to −19.2%. The effective cooperation rate was 92.0% (92/100) in the study group and 62.0% (62/100) in the control group; the agitation incidence was 8.0% (8/100) in the study group and 38.0% (38/100) in the control group. The agitation incidence was 8.0% (8/100) in the study group and 38.0% (38/100) in the control group. The initial sedation insufficiency rate (UMSS score < 3) in the study group was 8.0% (8/100), and all cases achieved effective sedation after rescue dosing; no sedation insufficiency or failure was found in the control group.

**Table 3:** Comparison of intraoperative cooperation and incidence of agitation between the two groups of children.

Group	*n*	No agitation	Mild agitation	Moderate agitation	Severe agitation	Effective cooperation rate	Agitation incidence rate
Study group	100	64 (64.0%)	28 (28.0%)	8 (8.0%)	0 (0.0%)	92 (92.0%)	8 (8.0%)
Control group	100	30 (30.0%)	32 (32.0%)	24 (24.0%)	14 (14.0%)	62 (62.0%)	38 (38.0%)

Compared with the control group, *P* < 0.01.

### 3.3 Safety evaluation

During the intraoperative and 30-minute postoperative observation periods, no serious adverse reactions such as respiratory depression, severe bradycardia, hypotension, or allergic reactions occurred in either group (95%CI for serious adverse reaction rate: 0.0% to 3.0%). One child in the study group experienced a brief slowdown in heart rate (down to 85 beats/min), which recovered spontaneously without special treatment (95%CI for bradycardia incidence: 0.0% to 5.4%). Two children in the control group had mild nausea without vomiting, and their symptoms resolved spontaneously after surgery (95%CI for nausea incidence: 0.2% to 6.9%). All children successfully completed the operation and were discharged normally.

## 4 Discussion

This retrospective randomized controlled trial systematically evaluated the effectiveness and safety of intranasal dexmedetomidine hydrochloride for preoperative sedation in 200 children aged 2 to 6 years undergoing cosmetic suturing of emergency facial trauma. The results showed that compared with routine comfort care alone, nasal instillation of dexmedetomidine hydrochloride significantly improved the effective intraoperative cooperation rate (92.0% vs. 62.0%, *P* < 0.01, 95%CI: 19.2% to 40.8%) and reduced the incidence of agitation (8.0% vs. 38.0%, *P* < 0.01, 95%CI: −40.8% to −19.2%), stabilized the intraoperative hemodynamic indicators (heart rate, mean arterial pressure) in children (95%CI for HR difference: −14.7 to −8.1; 95%CI for MAP difference: −5.8 to −3.2), with no serious adverse reactions observed, thus exhibited high safety.

Notably, the 5-point University of Michigan Sedation Scale (UMSS) adopted in this study is a validated and widely used tool for pediatric procedural sedation assessment (10). It is particularly suitable for evaluating intraoperative cooperation in children aged 2–6 years undergoing short-term emergency cosmetic suturing. Its clear operational definitions minimize inter-observer variability, ensuring the reliability of intraoperative cooperation evaluation. The mapping between our 4-level agitation classification and UMSS scores (severe agitation=UMSS 1–2, moderate agitation=UMSS 3, slight restlessness=UMSS 4, no restlessness=UMSS 5) is consistent with the clinical practice of pediatric sedation, further supporting the validity of our outcome indicators.

The initial sedation insufficiency rate in the study group was only 8.0% (8/100), and all cases achieved effective sedation after standardized rescue dosing without any true sedation failure requiring alternative sedatives. This further confirmed the high clinical reliability and controllability of dexmedetomidine as a sole sedative agent for pediatric emergency cosmetic suturing. Consequently, all children in the study group completed the cosmetic suturing procedure successfully. Preschool children have weak safety awareness, are lively and active, and are prone to accidental injuries leading to facial trauma (11). The accuracy of traditional debridement and suture alignment is insufficient, and postoperative scars are obvious, which not only affects facial beauty, but may also cause long-term psychological trauma to children, which is not conducive to healthy mental development (12). These research results confirm the clinical application value of intranasal dexmedetomidine hydrochloride in cosmetic suturing of emergency facial trauma in children, and provide evidence-based support for solving the clinical problem of poor cooperation in children's fine cosmetic suturing operations.

Poor intraoperative cooperation is the core clinical difficulty in cosmetic suturing of emergency facial trauma in children, which is consistent with the results of previous studies. It has been reported that children often show significant resistance during invasive medical procedures, seriously affecting the accuracy of the surgery (13). The incidence of agitation in the control group in this study was 38.0%, further verifying the common clinical problem of insufficient cooperation during emergency cosmetic suturing in children without sedation intervention. The significant improvement in cooperation and reduction in agitation in the study group are closely related to the unique sedative properties of dexmedetomidine hydrochloride: as a highly selective *α*2-adrenergic receptor agonist, it acts on the locus coeruleus, inhibits the release of norepinephrine, produces a natural non-rapid eye movement sleep state, and achieves “awakenable sedation” (14, 15). In this study, the UMSS Sedation Scale was used for objective sedation assessment, which eliminated subjective observation bias and ensured the reliability and accuracy of the sedation effect evaluation. This sedative state makes it easy for children to wake up and cooperate with simple instructions, which is especially suitable for short-term emergency cosmetic suture surgeries. In addition, the intranasal route of administration has the advantages of non-invasiveness, simple operation, and quick onset of action (about 10 min in this study). It is highly acceptable to children and their families and avoids the psychological resistance caused by venipuncture or enema, thus further improving children's cooperation ability (16).

In this study, the intraoperative heart rate and mean arterial pressure of the study group were significantly lower than those of the control group, but all indicators were within the normal physiological range, which is consistent with the research results of Wang et al. (14). This mild hemodynamic change was the result of dexmedetomidine hydrochloride stimulating central and peripheral *α*2-adrenergic receptors, inhibiting sympathetic activity, and enhancing vagal activity. This stabilizing effect has important clinical significance for emergency facial trauma cosmetic suturing: on the one hand, it reduces the increase in blood pressure and wound bleeding caused by agitation in children, ensures a clear surgical field of view, and improves the accuracy of layered tension-reducing suturing; on the other hand, it reduces the cardiovascular stress response of children and improves the safety of surgery in emergency situations.

It is worth noting that the most prominent advantage of dexmedetomidine hydrochloride over traditional sedative drugs is that it has minimal impact on respiratory function. The blood oxygen saturation of all children in this study remained above 95%, and no respiratory depression occurred in either group, which is consistent with the results of Zheng et al. (16) and Giordano et al. (17). This feature is particularly important in pediatric emergency trauma patients because they are often satiated and at high risk for respiratory depression leading to regurgitation and aspiration. Only 1 child in the study group experienced transient bradycardia that recovered spontaneously, and 2 children in the control group experienced mild nausea, indicating that intranasal dexmedetomidine hydrochloride has a good safety profile in this population.

In addition to its sedative effect, dexmedetomidine hydrochloride also has a significant synergistic analgesic effect, which is an important reason for improving the intraoperative cooperation of children in this study. It is confirmed that dexmedetomidine hydrochloride can inhibit nociceptive signal conduction through the dorsal horn of the spinal cord and reduce the release of inflammatory mediators, thereby exerting analgesic and anti-inflammatory effects. In this study, the study group received lidocaine local anesthesia combined with intranasal instillation of dexmedetomidine hydrochloride. The children's pain response was significantly reduced, agitation caused by pain was avoided, and the smooth progress of fine cosmetic suture surgery was ensured (18, 19). It has also been reported that adding dexmedetomidine hydrochloride to local anesthetics can enhance the analgesic effect and reduce the dosage of anesthetics, which is consistent with the results of this study (20, 21). Relevant studies have shown that dexmedetomidine, as an auxiliary analgesic, can reduce the dosage of other narcotic analgesics during surgery, reduce the risk of adverse reactions, and speed up postoperative recovery. This is consistent with the results of this study that the children in the study group recovered well after surgery and had no obvious adverse reactions (22). The precise and stable surgical environment created by sedation and analgesia is the key to improving the quality of cosmetic sutures, reducing scar formation, and improving family satisfaction. It is the core goal of cosmetic sutures for children's emergency facial trauma.

Traditional pediatric emergency sedation methods have obvious limitations: chloral hydrate enema has poor acceptability and may cause nausea, vomiting and unstable sedation due to anal drug discharge; midazolam (oral/intravenous injection) has a high risk of respiratory depression and abnormal excitement, especially in young children. In contrast, intranasal dexmedetomidine hydrochloride has multiple advantages: non-invasive route of administration and high acceptability; rapid onset of action and stable sedative effect; minimal impact on respiratory function and high safety; synergistic analgesic effect, reducing the need for additional analgesics. These advantages make it more suitable for the emergency and short-term characteristics of cosmetic suturing of children's facial wounds.

This study still has certain limitations: first, it is a single-center study with a limited sample size, and there may be a certain selection bias; second, the follow-up time is short, and the long-term cosmetic effect of facial scars and the long-term safety of the drug were not evaluated; third, this study only set up a conventional placebo care control group and did not directly compare it with other commonly used sedative drugs (such as midazolam, chloral hydrate).

Multi-center, large-sample randomized controlled trials should be carried out in future studies to further verify the effectiveness and safety of the drug; long-term follow-up should be added to evaluate the long-term cosmetic effect of scars and family satisfaction; the clinical advantages of dexmedetomidine hydrochloride should be compared with traditional sedative drugs; in addition, the optimal dose, timing and method of intranasal dexmedetomidine hydrochloride for children of different ages and weights need to be further explored. With the development of new drug delivery technologies (such as dexmedetomidine hydrochloride microneedle patches), the acceptability and clinical effects of drugs are expected to be further improved, which will provide more optimized sedation options for pediatric emergency clinics.

## 5 Conclusion

Dexmedetomidine hydrochloride preemptive sedation was used to prepare children for emergency facial trauma cosmetic suture surgery. It could significantly improve children's intraoperative cooperation, significantly reduce the incidence of agitation, and stabilize vital signs such as heart rate and mean arterial pressure during the operation. It was safe and had no obvious respiratory depression or serious adverse reactions. The drug was easy to administer through the nose, was non-invasive, and had rapid onset of action. It was particularly suitable for the sedation needs of children in emergency short-term surgeries. It could create favorable conditions for fine cosmetic suturing during the operation, thereby improving the effect of surgical treatment and family satisfaction, and had high clinical promotion value. With the continuous development of new drug delivery technologies, the application prospects of dexmedetomidine in the field of children's sedation would be broader.

## Data Availability

The original contributions presented in the study are included in the article/supplementary material, further inquiries can be directed to the corresponding author.
